# Prognostic Impact of the Advanced Lung Cancer Inflammation Index (ALI) in Metastatic Non-Small Cell Lung Cancer Treated with First Line Chemotherapy

**DOI:** 10.31557/APJCP.2021.22.4.1149

**Published:** 2021-04

**Authors:** Chaichana Chantharakhit, Nantapa Sujaritvanichpong

**Affiliations:** *Division of Medical Oncology, Department of Internal Medicine, Buddhasothorn Hospital, Chachoengsao, Thailand. *

**Keywords:** Prognostic factors, survival analysis, lung cancer, advanced lung cancer inflammation index (ALI)

## Abstract

**Background:**

The advanced lung cancer inflammation index (ALI) has been reported to predict the overall survival in patients with advanced non-small cell lung cancer (NSCLC). However, no previous studies have examined the prognostic significance of ALI in metastatic NSCLC treated with first line chemotherapy. The objective of this study was to explore the relationship between ALI and the prognosis of metastatic NSCLC treated with first line chemotherapy.

**Materials and Methods:**

Data of 109 metastatic NSCLC patients who had completed first line treatment with chemotherapy was collected. A multivariate flexible parametric proportional-hazards model with restricted cubic splines (RCS) was used to explore and identify the independent prognostic factors, including clinical potential factors and ALI for the overall survival. Multivariate regression analysis was used to evaluate the potential prognostic factors associated with short survival less than 6 months. The analysis of the restricted mean survival time (RMST) method was used to estimate the event-free time from zero to 18 months.

**Results:**

The median OS was 10.9 months (95%CI 9.57-13.18) and median PFS was 7.5 months (95%CI 6.85-8.00).The multivariate survival analyses revealed two prognostic factors for worse survival: Poor ECOG PS (HR46.90; 95%CI 2.90-758.73; p=0.007) and progressive disease after completing the first line chemotherapy treatment (HR 2.85; 95%CI1.18-6.88; p=0.02),whereas a low ALI <11 referred to a non-significant prognostic factor (HR 1.42; 95%CI 0.67-3.01; p=0.364).The results of the multivariate regression analysis revealed that the low ALI and progressive disease status were significantly associated with the short survival outcome (OR 5.12; 95%CI 1.11-23.65; p=0.037; OR 12.57; 95%CI 3.00-52.73; p=0.001).

**Conclusions:**

A low ALI was associated with the short survival in metastatic NSCLC treated with chemotherapy. However, using ALI as a prognostic factor only was still too limited. Other considerable clinical prognostic factors should also be used simultaneously, which would have strong significant prognostic impacts.

## Introduction

Nowadays, there are various medicines for treating advanced non-small cell lung cancer (NSCLC), e.g., platinum-base chemotherapy, targeted therapy, and immune checkpoint inhibitors. Choosing the appropriate treatment methods basically relies on personalized medicines in order to suit individual patients. Even so, it has been found that the same methods still produced different outcomes in each patient. Cancer treatment outcomes can also be evaluated in several forms, e.g., tumor response, disease free interval/progression free interval (Nishino et al., 2014; Shukuya et al., 2016), and overall survival which is the maximum goal for cancer treatment. Therefore, evaluation of cancer treatment efficiency gives precedence to the overall survival as the first priority. Furthermore, there are some internal factors underlying patients (prognostic factors), i.e., clinical factors and biomarkers that can influence survival (Teramukai et al., 2009; Galvano et al., 2020).

Data from previous studies tried to find the prognostic factors that helped to predict the survival in treating patients with lung cancer; for example, the study of Kobayashi et al., (2019) in patients with lung cancer at stage 1A with surgical resection. It was found that gender, red cell distribution width (RDW), neutrophil-to-lymphocyte ratio (NLR), and advanced lung cancer inflammation index (ALI) were the key prognostic factors. A number of studies (Berardi et al., 2019; Salem et al., 2018; Phillipson and Kubes, 2011) also found that inflammation inside the body associated with tumor cell proliferation resulted in the progression of the disease afterwards because the NSCLC cells could release immunoreactive IL-8 and stimulate the release of polymorphonuclear neutrophils (PMNs) (De Larco et al., 2004; Masuya et al., 2001; Yuan et al., 2000). Mediate ARG1 release by PMNs can inhibit T-cell proliferation and favor tumor cell progression (Rotondo et al., 2009; Munder et al., 2005). Therefore, some data revealed that patients with high neutrophil had a worse prognosis than those with normal neutrophil (Wu et al., 2019; Shaul and Fridlender, 2017). As such, there are studies on the neutrophil to-lymphocyte ratio (NLR) reflecting the balance between the inflammation pathway activity and anti-immune function (Faria et al., 2016; Templeton et al., 2014). This is a key independent prognostic factor of NSCLC and is associated with the treatment response (Ren et al., 2019; Tomita et al., 2011). Nonetheless, the cut-off levels of NLR were still different in each particular study (Vano et al., 2018; Zhao et al., 2015). 

In addition, the issue of the albumin levels in the body has been used as a predictive and prognostic factor for survival. It was found that the levels of serum albumin in patients with lung cancer before treatment affected their survival outcome (Detsky et al., 1984; Espinosa et al., 1995). However, those levels referred in some studies were still different; for example, Yao et al., (2014) studied the treatment in 316 Chinese patients. The serum globulin albumin ratio over 0.58 could be used as a predictive and prognostic biomarker to predict the treatment and survival in NSCLC stage 4. However, the study focused on the total number of patients with lung cancer without separating the treatment methods. Likewise, Ikeda et al., (2017) used 3.40 g/dL of albumin to help predict the treatment in poor performance status NSCLC Japanese patients for chemotherapy or best supportive care because the albumin levels associated with their nutritional status affected the survival outcome in those with malnutrition. 

There is also the data of the body mass index (BMI), which revealed that reducing the BMI significantly affected the survival rates in both types of cancer, i.e., NSCLC and SCLC (Shepshelovich et al., 2019; Iyengar et al., 2016) leading ALI, which was a biomarker obtained from the calculation between the BMI (kg/m^2^) × albumin (g/dL)/neutrophil to-lymphocyte ratio (NLR) according to the theory (Jafri et al., 2013). Therefore, it is believed that a biomarker represents all biomarkers associated with the prognostic factors (Tomita et al., 2018; Wang et al., 2020).

Therefore, the key objective of this study was to examine only metastatic NSCLC patients who had completed first line chemotherapy in order to examine the factors influencing their overall survival and survival less than six months (short survival). The ability of the ALI as a prognostic factor impacting on this group of patients was also taken into account. The target was further narrowed down more than found in previous literature. Moreover, this was the first study conducted on Thai patients.

## Materials and Methods

Patients: The study was a time-to-event analysis with an ambi-spective observational cohort study design. Data were collected from metastatic NSCLC patients who had received first line chemotherapy at the Division of Medical Oncology, Buddhasothorn Hospital, Chachoengsao, Thailand from July 2016 to June 2020. The study protocol was approved by the Institutional Review Board of Buddhasothorn Hospital number BSH-IRB 054/2563.

Data collection: Data of the potential prognostic predictors were collected from NSCLC stage 4, i.e., gender, age, weight, height, site of the metastasis, brain metastasis, ECOG performance status, progression free interval, date of death, pretreatment neutrophil-to-lymphocyte ratio (NLR), level of the pretreatment albumin, first line treatment with chemotherapy, and second line treatment. Data of the date of death were explored and collected from the database of the Bureau of Registration Administration, Department of Provincial Administration, Ministry of Interior, Thailand. Only the cause of death due to lung cancer was brought for data analysis. Patients were followed up for their disease status until October 2020. Follow up time is measured from time zero (the date of start first line chemotherapy) until the event occurs, or the study ends. Patients who had started treatment at other hospitals or received incomplete chemotherapy were excluded. 

Statistical analyses: Statistical analyses were performed using the STATA version 16 (StataCorp, TX,USA) to compare the clinical outcomes according to the patients’ characteristics. A flexible parametric survival model of univariate and multivariate analyses was used to derive the prognostic model via the stpm2 package. The main advantage of this non-rigid parametric survival model, beyond the Cox regression, was its ability to estimate the baseline cumulative hazard function via the use of restricted cubic splines. Survival curves were also estimated using the Kaplan–Meier method. To identify the prognostic factors for short survival, multivariate regression analysis was conducted. Restricted means of survival time was demonstrated to estimate the event-free time at 18 months.

## Results

**Figure 1:**
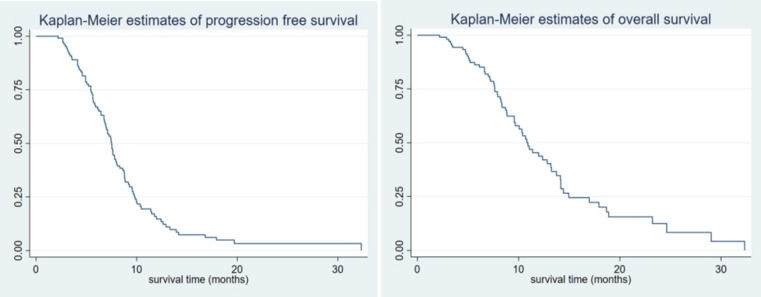
Kaplan-Meier Curve of the PFS and OS (mPFS 7.5 months, mOS 10.9 months)

All 109 patients were diagnosed with metastatic NSCLC and received complete first line treatment with chemotherapy. The sample comprised 72 males (66.1%) and 37 females (33.9%). The youngest was 38 years old, and the oldest was 83 years old with the average age being 60.58 years. The median follow-up of patients still alive was 8.49 months with median progression free survival (mPFS) 7.5 months (95%CI 6.85-8.00) and median overall survival (mOS) 10.9 months (95%CI 9.57-13.18) (Figure 1.) The basic data of the patients are displayed in Table 1.

**Figure 2:**
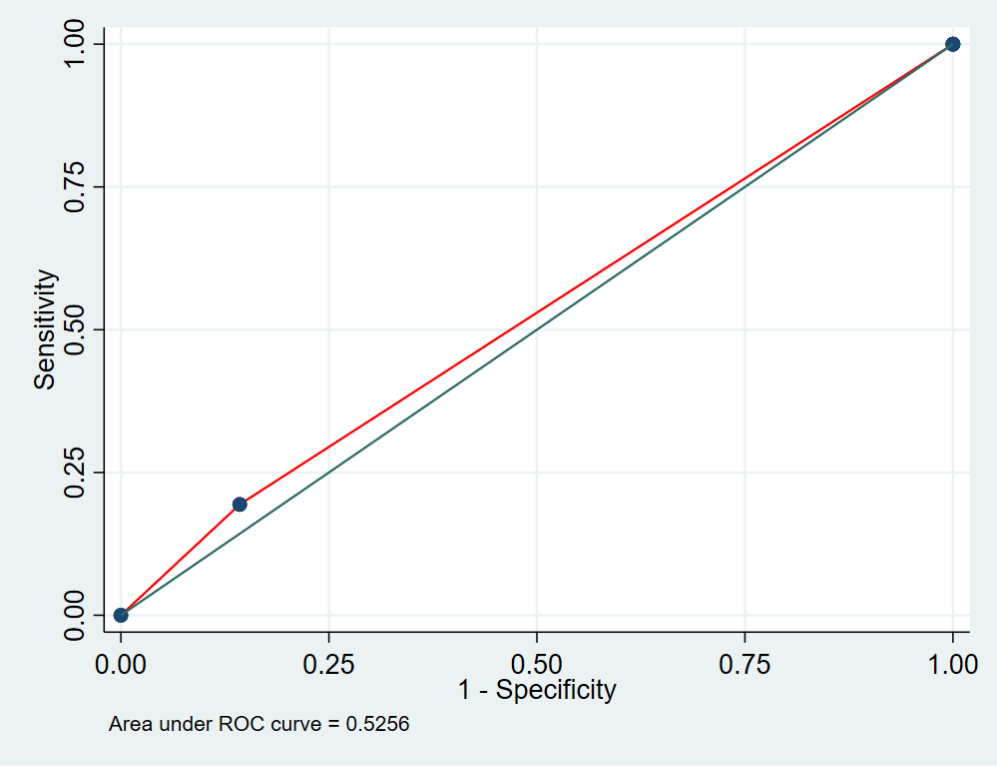
The Area under the Receiver Operating Characteristic Curve (ROC curve) of ALI when using the Cut-off Point <11 to Predict the Probability of Death, AUC 0.52 (95% CI 0.45-0.60).

The potential predictors associated with survival in metastatic NSCLC patients were analyzed, i.e., gender, elderly patients (age > 60 years), ECOG performance status (ECOG PS), tumor grading, anemia (hemoglobin < 12 g/dL), initial brain metastasis at diagnosis, response status after completing first line chemotherapy, received second line treatment, and ALI. The value <11 was used as the cut-off level because it was the point with the most discrimination (area under ROC 0.52; sensitivity 19.40%; specificity 85.71%; positive likelihood 1.36) (Figure 2.)

When analyzing the variables based on the univariate flexible parametric proportional-hazards model with restricted cubic splines (RCS), it was found that poor ECOG performance status, progressive disease after completing first line chemotherapy, and low ALI (<11) were the prognostic factors affecting the survival outcome. Received second line treatment had protective effects on the overall survival (Table 2).

Nevertheless, when analyzing the variables based on the multivariate flexible parametric proportional-hazards model with the restricted cubic splines (RCS), it was found that the poor ECOG performance status and progression of the disease after completing first line chemotherapy were the poor prognostic factors. Additionally, receiving second line treatment had protective effects on the overall survival (Table 3). However, when analyzing the variables affecting the short survival of less than six months by multivariate logistic regression analysis, it was discovered that the ECOG performance status 2 and received second line treatment were good prognostic factors with protective effects on the short survival. It was also found that the progression of the disease after completing first line chemotherapy and low ALI were poor prognostic factors on the short survival in patients receiving first line chemotherapy (Table 4).

When analyzing the low ALI as a prognostic marker by the RMST method at 18 months, it was established that when the confounder was adjusted, the mean difference of the event-free time was -0.92 months (95%CI -3.19; 1.35; p=0.427) (Table 5). When using the response status after first line chemotherapyas a prognostic marker when the confounder was adjusted, it was found that patients with the progression of the disease after completing first line chemotherapy had a mean difference of the event-free time =-2.89 months when compared with the partial response group (95% CI -5.52; -0.26; p=0.031) (Table 6).

**Table 1:** Patient Characteristics

Data		Number (%)
Gender	Male	72 (66.1)
	Female	37 (33.9)
Age	< 60 years	57 (52.3)
	> 60 years	52 (47.7)
ECOG Performance	0	16 (14.7)
	1	71 (65.1)
	2	20 (18.4)
	3	2 (1.8)
Cell type	Adenocarcinoma	97 (89)
	Squamous cell carcinoma	12 (11)
Tumor grade	Well differentiate	2 (1.8)
	Moderate differentiate	83 (76.2)
	Poorly differentiate	24 (22)
Anemia(Hb < 12 g/dL)		50 (45.9)
Initial brain metastasis		11 (10.1)
Response after first line chemotherapy	Partial response	65 (59.6)
	Stable disease	19 (17.4)
	Progressive disease	25 (23)
Received second line treatment		53 (48.6)

**Table 2:** Hazard Ratio of the Overall Survival by the Potential Predictors [Univariate Flexible Parametric Proportional-hazards Model with Restricted Cubic Splines (RCS)].

Potential predictors		uHR	95%CI of Hazard Ratio	P-value
Gender	Female	0.62	0.36-1.06	0.083
	Male	1	-	
Elderly (Age > 60 years)		0.93	0.57-1.52	0.773
ECOG PS	1	5.84	1.96-17.39	0.002*
	2	6.64	2.12-20.74	0.001*
	3	11.47	1.21-108.54	0.033*
	0	1	-	
Tumor grade	Moderate differentiate	1.68	0.39-7.20	0.482
	Poorly differentiate	2.86	0.61-13.33	0.182
	Well differentiate	1	-	
Anemia (Hb < 12 g/dL)		0.75	0.45-1.26	0.282
Initial brain metastasis		0.96	0.46-2.02	0.918
Response after first line chemotherapy	Stable disease	1.14	0.61-2.16	0.674
	Progressive disease	2.42	1.35-4.36	0.003*
	Partial response	1	-	
Received second line treatment		0.42	0.26-0.70	0.001*
Low ALI (<11)		1.93	1.02-3.64	0.042*

*Statistically significant p-values

**Table 3:** Hazard Ratio of Survival by Potential Predictors [Multivariate Flexible Parametric Proportional-Hazards Model with Restricted Cubic Splines (RCS)].

Potential predictors		mHR	95%CI of Hazard Ratio	P-value
Gender	Female	0.59	0.31-1.11	0.104
	Male	1	-	
Elderly (Age > 60 years)		1.08	0.54-2.14	0.827
ECOG PS	1	9.9	2.32-42.21	0.002*
	2	14.81	3.05-71.86	0.001*
	3	46.9	2.90-758.73	0.007*
	0	1	-	
Tumor grade	Moderate differentiate	0.52	0.08-3.45	0.5
	Poorly differentiate	0.72	0.11-4.80	0.733
	Well differentiate	1	-	
Anemia (Hb < 12 g/dL)		0.83	0.47-1.47	0.525
Initial brain metastasis		0.68	0.24-1.95	0.476
Response after first line chemotherapy	Stable disease	1.78	0.84-3.78	0.134
	Progressive disease	2.85	1.18-6.88	0.020*
	Partial response	1	-	
Received second line treatment		0.48	0.26-0.87	0.016*
Low ALI (<11)		1.42	0.67-3.01	0.364

*Statistically significant p-values

**Table 4:** Multivariate Logistic Regression Model for Short Survival of Six Months

Potential predictors		OR	95%CI of Hazard Ratio	P-value
Gender	Female	0.8	0.22-2.91	0.738
	Male	1	-	
Elderly (Age > 60 years)		2.78	0.81-9.52	0.104
ECOG PS	1	0.2	0.04-1.05	0.058
	2	0.06	0.01-0.54	0.011*
	3	1.74	0.03-104.15	0.792
	0	1	-	
Tumor grade	Moderate differentiate	0.94	0.23-3.81	0.933
	Poorly differentiate	1	-	
	Well differentiate	1	-	
Anemia (Hb < 12 g/dL)		1.4	0.40-4.85	0.599
Initial brain metastasis		1 (omitted)		
Response after first line chemotherapy	Stable disease	0.38	0.06-2.43	0.305
	Progressive disease	12.57	3.00-52.73	0.001*
	Partial response	1	-	
Received second line treatment		0.15	0.04-0.58	0.005*
Low ALI (<11)		5.12	1.11-23.65	0.037*

*Statistically significant p-values

**Table 5:** Event-Free Time (RMST) in Patients with ALI as a Prognostic Marker

Event free time (based on 18 months)	Low ALI (<11) (n= 19)	High ALI (>11) (n= 90)	Difference Mean	95%CI	P-value
RMST (month), Mean±SE					
Crude	9.13 ± 1.26	11.93 ± 0.56	-2.8	-5.50 ,-0.10	0.042*
Adjusted	10.77 ± 1.07	11.69 ± 0.43	-0.92	-3.19 , 1.35	0.427

*Statistically significant p-values

**Figure 3:**
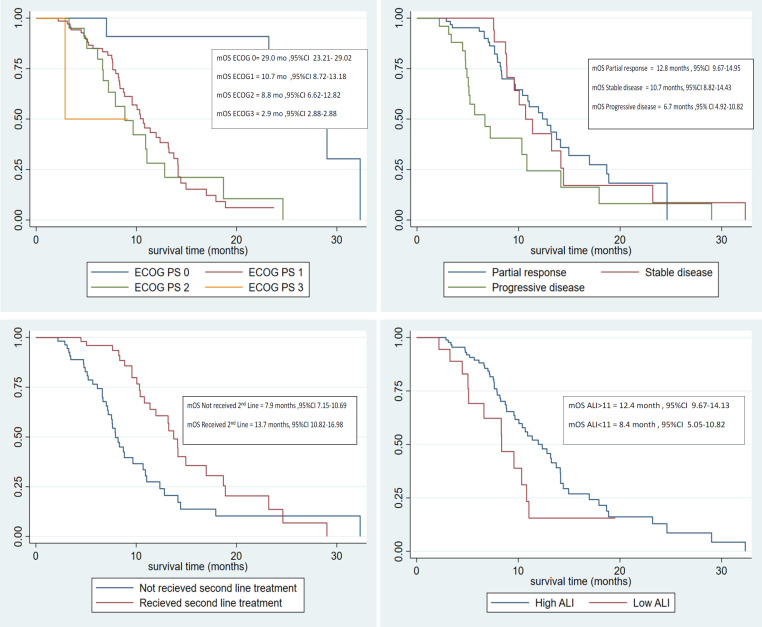
Kaplan-Meier Estimates of Overall Survival, According to Significant Prognostic Factors by Univariate Analysis

**Table 6:** Event-Free Time (RMST) in Patients with Response Status after First Line Chemotherapy

Event free time (based on 18 months)	RMST (month), Mean±SE (Univariable analysis)				RMST (month), Mean±SE (Multivariable analysis)			
	Crude	Difference			Adjusted	Difference		
		Mean	95%CI	P-value		Mean	95%CI	P-value
Partial response	12.42 ± 0.66	-	-	-	12.45 ± 0.64	-	-	-
Stable disease	12.40 ± 1.10	-0.02	-2.78 ,2.75	0.99	11.43 ± 0.65	-1.02	-2.95 , 0.92	0.303
Progressive disease	8.16 ± 0.99	-4.26	-6.60, -1.93	<0.001*	9.56 ± 1.02	-2.89	-5.52, -0.26	0.031*

*Statistically significant p-values

## Discussion

According to the results, it was found that increasing the ECOG performance status (poor ECOG performance status) and progression of the disease after completing the first line chemotherapy treatment were the two strongest prognostic factors for survival, whereas received second line treatment was the protective effect for survival. However, when considering the short survival less than six months, it was found that the progression of the disease after completing first line chemotherapy treatment and low ALI were strongly associated with the short survival, whereas received second line treatment and ECOG performance status 2 were strongly associated protective effects for the short survival in patients with chemotherapy.

The values of the cut-off level of ALI were still unclear (Ozyurek et al., 2018). Nonetheless, there was proof that low ALI affected the poor survival outcome resulting from a low ALI based on a decreased BMI, a lower Alb, and a high NLR that indicated a poor prognosis as well as high systemic inflammation. Moreover, the BMI levels might represent the nutritional status of the patients (Hua et al., 2019). All were biomarkers associated with poor survival in cancer. Similar to Tomita et al., (2018) who found that ALI < 37.66 was the predictive post-operative survival in patients with lung cancer who received surgery. Bacha et al., (2017) also discovered that ALI <18 was associated with poor prognosis in patients with metastatic lung cancer. However, Diaz De Teran Lopez et al., (2020) observed that the cut-off level at 37 was used in the metastatic stage. Most previous studies were overall studies on metastatic NSCLC patients without treatment methods, which should have actually been a key factor influencing the survival outcome. Different cancer staging was also a key factor on survival. Therefore, the cut-off levels of the ALI in each setting were different. 

According to the meta-analysis of Zhang et al., (2019) 1,587 patients with lung cancer were collected from eight retrospective cohort studies on ALI with different cut-off levels used. Additionally, the stages of the disease were also different. Each study contained a metastatic stage, mixed staging, and treatment with unseparated methods. It was found that pretreatment ALI <24.23 affected a poor survival outcome. Therefore, it could be concluded that low ALI was a poor prognostic factor in NSCLC patients. However, there were some limitations for prognosis in patients with different settings. 

This study used ALI <11 as the low ALI level because it was the point with the most discrimination from the area under the ROC curve. Nevertheless, for the multivariate survival analysis, no significant association with survival was found possibly due to the small population. However, when analyzing the association with a short survival less than six months, it was discovered that ALI <11 was significantly associated with the short survival in patients with lung cancer receiving chemotherapy. 

Another strong poor prognostic factor uncovered in this study was the progression of the disease after completing the first line chemotherapy treatment, which was significantly associated with poor survival outcome and short survival less than six months. Average event-free survival time by the RMST method was found that patients with the progression of the disease after completing the first line chemotherapy had significant difference when compared with the partial response group, while ALI had no statistically significant difference event-free time between low and high ALI. Furthermore, the negative ECOG performance status could be a predictor of a poor prognosis. An interesting item of data revealed that patients with lung cancer that had an ECOG performance status 2 would significantly have protective effects on the short survival after chemotherapy. 

It was also revealed that among patients with lung cancer who had received first line chemotherapy, half of them (48.6%) would survive and would be strong enough to receive second line treatment. Therefore, receiving second line treatment was significantly regarded as a protective effect on the survival outcome in lung cancer treatment. Nevertheless, those with the initial presentation of brain metastasis (10%) did not affect the poor prognosis on survival in this study.

However, although these results need further studies conducted on a larger population to validate the prognostic role of inflammatory biomarkers, especially the ALI, the benefits of the data from this study, both the inflammatory biomarker and clinical prognostic factors, could help plan the treatment, follow-up, and prognosis on survival in metastatic NSCLC patients who had received chemotherapy. This group of patients were the most common in clinical practice.

In conclusion, low ALI was associated with the short survival in metastatic NSCLC patients who had received chemotherapy. Although this study could not significantly demonstrate the predictors of the poor survival outcome of low ALI, possibly due to the small population, and thus no statistical power was seen, using ALI as a prognostic factor only was still too limited. Other considerable clinical prognostic factors should also be used simultaneously, e.g., poor ECOG performance status and response status after completing first line chemotherapy, which would have strong significant prognostic impacts.

## Author Contribution Statement

Chaichana Chantharakhit: designed the study, collected data, analyzed data, edited the final version Nantapa Sujaritvanichpong: collected data, reviewed the paper All author reads an approved the final version.
